# A smartphone-based intervention for young people who self-harm (‘PRIMARY’): study protocol for a multicenter randomized controlled trial

**DOI:** 10.1186/s12888-023-05301-x

**Published:** 2023-11-14

**Authors:** Anouk Aleva, Tessa van den Berg, Odilia M. Laceulle, Marcel A. G. van Aken, Andrew M. Chanen, Jennifer K. Betts, Christel J. Hessels

**Affiliations:** 1 HYPE Centre of Expertise on Early Intervention for Borderline Personality Disorder, GGz Centraal, Amersfoort, The Netherlands; 2 Department of Developmental Psychology, Utrecht University, Utrecht, The Netherlands; 3 Orygen, Melbourne, Australia; 4 Centre for Youth Mental Health, The University of Melbourne, Melbourne, Australia

**Keywords:** Self-harm, Young people, Randomized controlled trial, Smartphone-based intervention, ESM, Personality disorder

## Abstract

**Background:**

Self-harm in young people is a public health concern connected with severe mental health problems, such as personality pathology. Currently, there are no specific evidence-based interventions available for young people who self-harm. Therefore, we developed *PRe-Intervention Monitoring of Affect and Relationships in Youth (PRIMARY)*, a smartphone-based intervention, co-designed by clinicians and young people with lived experience of mental ill-health. PRIMARY combines the Experience Sampling Method (ESM) with weekly report sessions. The study aims to examine the effectiveness of PRIMARY with regard to reducing self-harm, and improving emotion regulation and quality of relationships.

**Methods:**

This study is a multicenter, parallel groups, randomized controlled trial (RCT) comparing the PRIMARY intervention to a waiting list control group. PRIMARY comprises 28 consecutive days of questionnaires five times each day (i.e., ESM) and four weekly report sessions. Participants will comprise 180 young people referred for treatment to the participating Dutch mental healthcare institutions and (1) are aged 12 to 25 years, and (2) engaged in ≥ 1 act of self-harm in the past year. Participants are randomly allocated to a study group after screening in a 1:1 ratio by an independent researcher using computer-generated randomization sequences with stratified block randomization by age (12 to 15 years / 16 to 25 years). Staff will conduct assessments with all participants at baseline (Wave 1), after 28 days (Wave 2), and in a subsample after 10 weeks of subsequent specialized treatment (Wave 3). The primary outcomes are self-harm, emotion regulation, and quality of relationships. Secondary outcomes include patient and clinician satisfaction. Exploratory analyses of ESM data will examine the relationship between emotions, social relationships, and self-harm.

**Discussion:**

The results of this trial will clarify whether an innovative smartphone-based intervention is effective for reducing self harm and improving emotion regulation and the quality of social relationships. It has the potential to fill a treatment gap of interventions specifically targeting self-harm. If proven effective, it would provide an accessible, easy-to-implement, low-cost intervention for young people. Furthermore, the ESM-data will allow detailed analyses into the processes underlying self-harm, which will contribute to theoretical knowledge regarding the behavior.

**Trial registration:**

ISRCTN42088538 (10.1186/ISRCTN42088538), retrospectively registered on the 26th of October 2022.

**Supplementary Information:**

The online version contains supplementary material available at 10.1186/s12888-023-05301-x.

## Background

Self-harm is common in young people and represents a major public health challenge [1–3]. Self-harm is defined as all intentional acts of self-injury or self-poisoning, irrespective of the type of motive [4]. Self-harming behavior is associated with problems in emotion regulation [5], enduring psychosocial problems [6], and shows a clear connection to severe mental health problems, such as depression, anxiety, substance use, personality pathology and suicidality [7–9]. Hence, early detection and intervention for self-harm are crucial. However, treatment for self-harm is often only available within specialist, psychotherapeutic programs, such as Dialectic Behavioral Therapy (DBT) and Mentalization Based Treatment (MBT), which target a range of difficulties [10, 11]. These programs entail complex and lengthy interventions, requiring extensive staff training and financial resources [12]. Hence, there is a need for simpler and ‘scalable’ evidence-based interventions that are specifically designed for self-harm. Such interventions might be more easily implemented across the healthcare system [13], making them available to more young people [12], and are likely to be more cost-effective [14].

### Self-harm

Beyond the immediate adverse effects of self-harm, like tissue damage [15], self-harm is a marker for an increased risk of developing various mental health problems [16, 17]. Given that the chance of repetition of self-harm within 12 months after the first incident increases with every prior act, with up to 70% in the case of four previous acts [18], it is important to intervene as early as possible to prevent persistent and profound problems. severe pathology. Recurrent self-harm is associated with severe psychopathology, such as borderline personality disorder (BPD) [19]. Studies have shown that over 40% of young people who self-harm during adolescence and have received mental healthcare, go on to receive a diagnosis of BPD in adulthood [20]. Therefore, interventions aimed at diminishing self-harm might also function as early intervention for those at risk of developing severe psychopathology, by targeting underlying problems of emotion regulation and interpersonal functioning [21].

Even though evidence-based treatments specifically aimed at preventing the recurrence of self-harm in young people engaging in the behavior are not yet available [22], positive effects of broader treatment programs provide important insights into the mechanisms underlying the reduction of self-harm. First, actions aimed at long-term improvement of psychosocial functioning have been found to be beneficial (e.g., re-orienting to a more positive future, reassessing one’s place in the social world; [23]. Second, actions aimed at breaking the association between an individual’s psychological or social state and the act of self-harm, i.e. ‘breaking the chain’, are important for short term change [23]. This includes strategies to manage immediate thoughts and feelings when the urge to self-harm arises, including improving emotion regulation, family support, and self-esteem [24]. Preceding the employment of such strategies, and essential to change harmful behavior, is gaining insight or awareness of problem areas and triggers [25, 26]. As the median time between the first thought of self-harm and the actual act has been found to be 35 h in some groups [27], there seems to be a ‘window of opportunity’ for the young person to recognize the separate steps of the ‘chain’ leading up to the act of self-harm. Subsequently, suggestions for more helpful coping techniques might guide the individual towards alternative strategies. Given that self-harm often fulfills the function of regulating emotions and interpersonal contexts [21], enhancing a young person’s insight into these areas seems especially important.

### Smartphone-based intervention

Help-seeking among young people is impeded by stigma, embarrassment, trouble recognizing symptoms, and a preference for self-reliance [28]. When young people do seek treatment, they are often faced with long waiting times within the mental healthcare system, which can demotivate them [29, 30]. This is especially problematic in the context of self-harm, given the direct and long-term adverse effects of the behavior. A smartphone-based intervention for young people who self-harm might contribute to overcoming the constraints of the current system, such as limited availability of clinicians [31]. Such an intervention would be self-directed and readily accessible for young people in their day-to-day lives.

The Experience Sampling Method (ESM) is a smartphone-based approach that asks people to fill out questions via their smartphone on multiple occasions during the day, for example regarding their feelings and behaviors [32]. ESM has been used to study the mechanisms underlying self-harm, which has led to the insight that heightened negative affect typically precedes the behavior [27, 33]. The ESM-methodology has thus far primarily been employed as a research tool for assessing self-harm. However, the act of systematically monitoring and registering feelings and behaviors might also be an intervention in and of itself, as it promotes reflection and facilitates the discovery of patterns of feelings and behavior [34]. The application of ESM as an intervention has already been recognized in the field of depressive disorders, where ESM is now studied as an add-on intervention to outpatient treatment programs [35, 36]. More recently, the use of ESM as a stand-alone smartphone-intervention has been demonstrated to be associated with positive mental health outcomes in young people in the community [37].

ESM has been posited as a promising framework for an intervention targeting self-harm [38]. Preliminary evidence suggests a possible decline in the frequency of the behavior when using smartphone-based interventions [39]. However, there is large variation in the type of smartphone-based interventions available and randomized controlled trials (RCTs) are needed to rigorously examine their effectiveness. Furthermore, co-designing an ESM-intervention in partnership with people with lived experience of mental ill-health enhances the fit of the intervention to the target group [40].

We have developed *PRe-Intervention Monitoring of Affect and Relationships in Youth* (PRIMARY), which is a smartphone-based intervention for young people who self-harm. PRIMARY combines ESM with brief, weekly, one-on-one structured report sessions where the young persons’ ESM-responses are discussed. This paper presents the study protocol for a multicenter, randomized controlled trial (RCT), evaluating the effectiveness of the PRIMARY intervention compared with a waiting list control. If PRIMARY is found to be effective in enhancing emotion regulation and reducing self-harm, it would offer an accessible, easy-to-implement intervention for young people throughout the mental healthcare system.

### Objectives

The first objective of this RCT is to determine whether PRIMARY, compared to a waiting list control, is effective in improving the following outcomes: self-harm, emotion regulation, and quality of relationships. To this end scores at baseline (Wave 1) and at the end of the intervention phase (Wave 2) will be compared. The second objective is to examine the effectiveness of PRIMARY combined with subsequent specialized treatment in the early intervention program Helping Young People Early (HYPE) [41] in improving the outcomes for young people with borderline personality pathology. Scores on the specified outcome variables after 10 weeks of HYPE treatment (Wave 3) will be compared to scores at baseline (Wave 1) and at the end of the intervention phase (Wave 2) for participants who attended HYPE. The third objective is to investigate the associations between emotions, relational support and conflict, adaptive coping strategies, and self-harm using individual-level ESM data. The fourth objective is to examine the participants’ and clinicians’ satisfaction regarding the use of PRIMARY. This will be examined at the end of the intervention phase (Wave 2).

## Methods

### Design

To facilitate transparency, the current protocol paper was written in accordance with the SPIRIT guidelines [42]. The SPIRIT Checklist is provided in the Supplemental Material.

This study is a multicenter, parallel groups, randomized controlled trial of the PRIMARY intervention compared with a waiting list control in outpatients aged 12 to 25 years (inclusive). The study also includes a nested evaluation of the effect of PRIMARY on the effectiveness of subsequent specialized treatment (HYPE) [41].

The design of the PRIMARY study was co-created by clinicians, young people with lived experience of mental ill-health, and researchers to ensure it is acceptable to all. For example, young people with lived experience provided feedback on how to adapt the ESM lay-out and script for the weekly report sessions to appeal to their age group. To guarantee the intervention is easy-to-implement, it is constructed to be delivered by research assistants (RA’s) rather than clinicians. RA’s hold a bachelor’s diploma in psychology or a related field and completed a suicide prevention e-learning module. Adherence to the prescribed PRIMARY intervention is promoted via detailed study procedures and scripts, as well as routine joint appointments with the RA, participant and AA or TvdB. All individual participant data (i.e., questionnaires and weekly, graphical reports) are made available within the electronic patient record of the participant. This is a direct benefit of participation in the study for the participant, as this information can be integrated into their diagnostic or treatment process by their clinician.

### Sample and setting

Young people aged 12 to 25 years (inclusive), with varying psychological problems, will be recruited after referral to one of the two participating mental healthcare institutions in the Netherlands: GGz Centraal and Mondriaan. These young people are awaiting further care at the institutions. Study inclusion criteria are: (1) engagement in at least one act of self-harm in the past year, and (2) sufficient understanding and skills in the Dutch language. In order to reflect ‘real world’ clinical practice, there are no exclusion criteria regarding participation in the study; participants can receive other interventions if judged necessary by the clinician in charge, also during participation in the study.

### Recruitment procedure

**Fig. 1:**
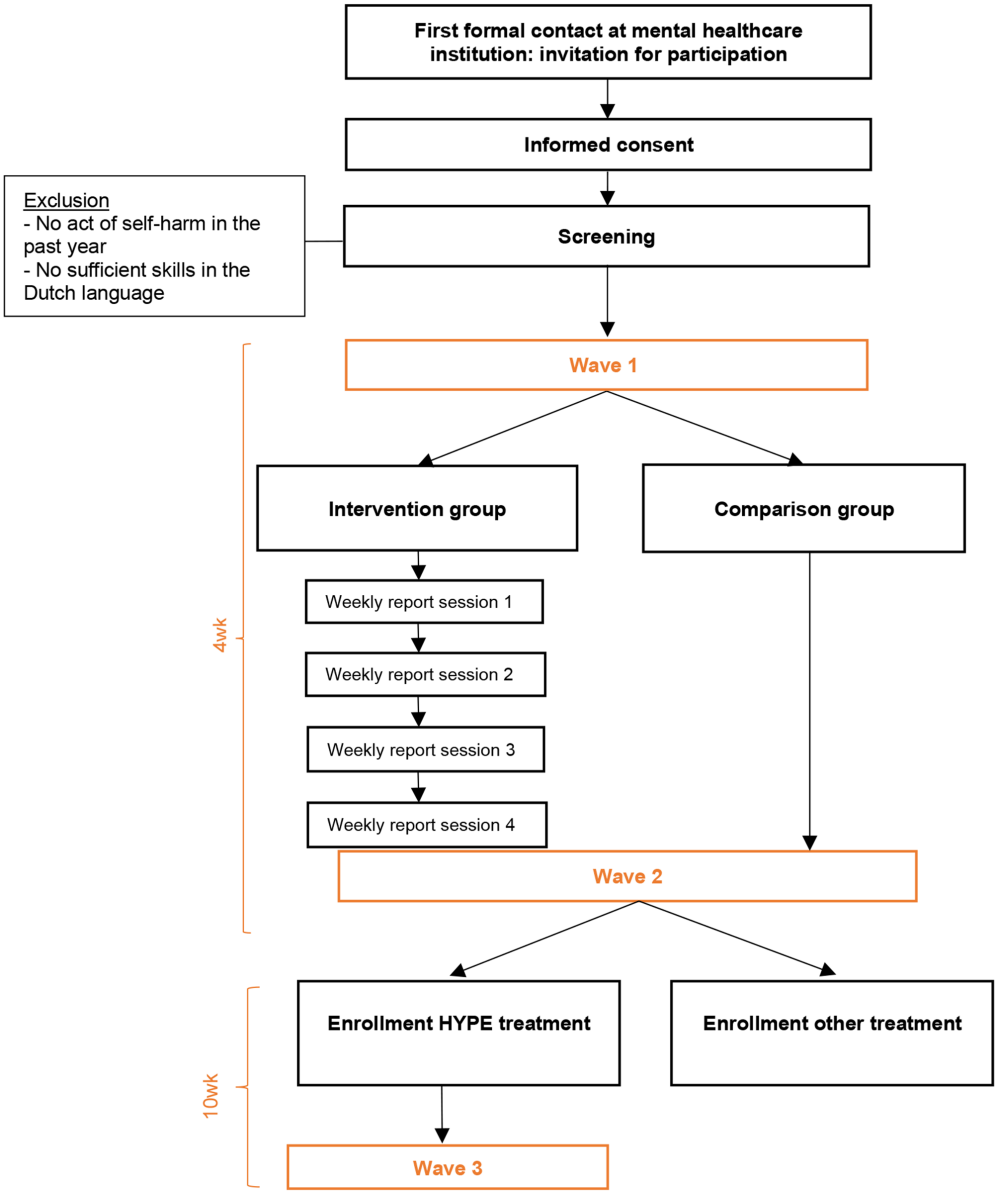
Flow diagram of the study design

Figure 1 depicts a flow diagram of the study design. A feasibility study was conducted to ensure an optimal design and recruitment procedure. Adjustments were made based on interviews with the participants. A description of the feasibility study can be found in the Supplemental Material. In this paper, the finalized procedures are described.

Young people receive an information letter about the PRIMARY study from the clinician or RA during their first formal contact at the mental healthcare institution (e.g., screening or intake session). After a two-day consideration period, an RA contacts the young person to further discuss participation and answer any questions. If the young person chooses to participate, written informed consent is obtained (also from parents or legal guardians for those aged < 16 years). A copy of the consent form is included in the Supplemental Material. Next, the young person fills out a brief screening questionnaire to confirm eligibility for the trial. If inclusion criteria are met, the baseline assessment (Wave 1) is scheduled with the RA, after which the young person is randomly assigned to a study group. At the end of the baseline assessment, the participant, independent of study group, can choose between two selected charities for the study to donate €5,00 to for their participation.

### Randomization and group allocation

Once eligibility is confirmed during screening, participants are randomly assigned to the intervention or the comparison group in a 1:1 ratio by an independent researcher using computer-generated randomization sequences with stratified block randomization by age (12 to 15 years / 16 to 25 years). Staff will not be blind to group allocation. Participants are blind to their allocated group during Wave 1 assessment. The nature of the intervention precludes concealment from participants and staff. Recruitment and retention in the trial will be maximized via study-specific training of RA’s and weekly staff-meetings to discuss the progress of the trial. In addition, retention was considered in great detail when designing the study by integrating input from young people with lived experience with mental-ill health, conducting a feasibility study and integrating recommended adjustments to increase compliance, and implementing insights from earlier ESM-studies.

### Study groups

#### Intervention group

After baseline assessment (Wave 1), the intervention group starts with the PRIMARY intervention. PRIMARY is an intervention consisting of two elements: ESM and weekly, one-on-one structured report sessions based on the individual’s ESM-answers. Participants fill out the ESM-questionnaire via their smartphone five times a day for 28 consecutive days (i.e., 4 weeks). Each ESM-questionnaire appears at random times within blocks of 2.5 h periods. Participants can choose a starting time for the first block between 06:00 AM and 12:00 PM. To encourage compliance with the ESM-questionnaire protocol, motivating text messages are sent once a week. In case compliance drops below 70% during the previous three days, participants receive an extra text message to stimulate respondence (maximum of once a week). If a participant allocated to the intervention group does not have a smartphone or internet on their smartphone, they can borrow a smartphone from the research staff. The internet on this smartphone is limited to accessing the ESM-questionnaire and therefore does not change the participant’s situation regarding social interactions.

The weekly report session encompasses a 15-minute video call (or appointment at the institution on request of the participant) with the RA over the course of the ESM-procedure (i.e., four times). During these sessions, the answers provided on the ESM-questionnaire over the past week are discussed in a structured, scripted way using a graphical report in which the outcomes are summarized. These sessions aim to enhance the participant’s insight into the connection between their emotions, activities and social context.

#### Comparison group

After baseline assessment (Wave 1), participants in the comparison group receive care-as-usual, which entails a waiting list for further care.

### HYPE

After finishing the assessment at the end of the study intervention phase (i.e. Wave 2), it is anticipated that participants in an early stage of BPD (i.e., ≥ three BPD criteria) will receive specialized treatment called HYPE (Helping Young People Early) [41]. Allocation to HYPE is determined by clinicians independent from the study. HYPE is a time-limited integrative treatment program based on the principles of Cognitive Analytic Therapy (CAT). The time-limit comprises 16 individual, weekly psychotherapy sessions, supplemented with psychiatric, psychosocial and family sessions if indicated. There is a follow-up period of six months, wherein the young person can schedule up to four sessions.

### Measures

All study measures and their timing during the study are depicted in Table 1. All participants, independent of study group, fill out questionnaires at baseline (Wave 1) prior to commencing the study intervention, and at the end of the intervention phase (i.e., four weeks later; Wave 2). Participants who subsequently enroll in HYPE also fill out the questionnaires 10 weeks after starting their treatment (Wave 3). All assessments other than the ESM-questionnaire are administered to both the intervention and comparison group at the mental healthcare institution or through a video call with an RA, depending on the preferences of the participant.

**Table 1:** Schedule of outcome measures

Construct	Measure	Screening	Wave 1	Intervention Phase	Wave 2	Wave 3
ESM-questionnaire*	Developed for study			X		
***Main study parameters***						
Self-harming thoughts and behaviors	Developed for study	X				
Emotion regulation	DERS-18		X		X	X
Quality of relationships	NRI-BSV		X		X	X
***Secondary parameters***						
Evaluation of PRIMARY*	UMUX Modified CSQ-8				X	
	Questions developed for the study					
***Other parameters***						
Demographics	Developed for study		X			
Concomitant treatment	Developed for study		X		X	X
BPD symptoms	SCID-II BPD PQ	X				
BPD behavior	BSL-23		X		X	X
Achievement of developmental milestones	DML		X		X	X
Life experiences	Modified LES		X		X	
Psychopathology	SPSY		X		X	X
Alternative Model for Personality Disorders	LPFS-BF PID-5-BF		X		X	X
Impulsivity	S-UPPS		X			
Internalizing problems	DASS		X		X	X
Loneliness	De Jong Gierveld questionnaire		X		X	X
Motivation for change	MYTS		X		X	X
Quality of life	ReQoL		X		X	X
Impact of COVID-19	Developed for study		X		X	X

*Only filled out by participants in the intervention group

ESM = Experience Sampling Method, DERS-18 = Difficulties in Emotion Regulation Scale, NRI-BSV = Network of Relationships Inventory – Behavior Systems Version, UMUX = Usability Metric for User Experience, CSQ = Client Satisfaction Questionnaire, SCID-II BPD PQ = BPD section of the Structured Clinical Interview for DSM-IV Axis II disorders Personality Questionnaire, BSL-23 = Borderline Symptom List, DML = Developmental Milestones List, LES = Life Experiences Survey; SPSY = Screeningsinstrument Psychische Stoornissen, LPFS-BF = Levels of Personality Functioning Scale 2.0 Brief Form, PID-5-BF = Personality Inventory for DSM-5 Brief Form, S-UPPS = UPPS-P impulsive behavior scale short version, DASS = Depression Anxiety Stress Scales, MYTS = Motivation for Youth Treatment Scale, ReQoL = Recovering Quality of Life

#### ESM-questionnaire

An ESM-questionnaire with 22–25 questions was developed for the purpose of the PRIMARY study. Recommendations to enhance the feasibility of an ESM-intervention in young people were taken into account in this process, like tailoring the first assessment to correspond to the young person’s sleep schedule [43]. The questionnaire is included in the Supplemental Material.

The daily ESM-questionnaire covers activities, relational support and conflict, emotions, substance use and, at the end of the day, functioning. The questions regarding emotions were informed by similar research [44] and the Positive And Negative Affect Scale (PANAS) [45]. One question regarding applied adaptive emotion regulation strategies is included, informed by Safety Planning Intervention [46]. Additionally, a question on self-harming thoughts and behaviors since the last notification is included. Answers are provided via a multiple choice format or on a Likert-scale ranging from 1 (*not at all*) to 7 (*very much*).

#### Main study parameters

**Self-Harming Thoughts and Behaviors.** Five questions were formulated to assess frequency and severity of self-harm, these are included in the Supplemental Material.

**Emotion Regulation.** The 18-item Difficulties in Emotion Regulation Scale (DERS-18) [47] is included. The DERS-18 comprises six subscales: ‘lack of emotional awareness’, ‘lack of emotional clarity’, ‘difficulty engaging in goal-directed behavior’, ‘impulse control difficulties’, ‘nonacceptance of emotional responses’, and ‘limited access to emotion regulation strategies’. Items are answered on a 5-point Likert scale. The scale has adequate construct and predictive validity, and good test-retest reliability [47].

**Quality of Relationships.** Participants fill out the 11-item version of the Network of Relationships Inventory – Behavior Systems Version (NRI-BSV) [48] regarding one parent and a best friend. The NRI-BSV comprises two subscales: ‘support’ and ‘negative interactions’. Items are answered on a 5-point Likert scale. The questionnaire was validated and the factor structure was confirmed [48].

#### Secondary parameters

**Evaluation of PRIMARY.** Participants in the intervention group rate usability of the ESM-element of PRIMARY on the 3-item Usability Metric for User Experience (UMUX) [49] on a 7-point Likert scale. This questionnaire correlates well with the original, longer version, is reliable, and the items load on one underlying factor [49]. Overall satisfaction with PRIMARY is indicated via a modified 4-item version of the Client Satisfaction Questionnaire-8 (CSQ-8) [50].

Included in the Supplemental Material are the modified version of the CSQ-8, two additional items regarding the duration and frequency of the ESM-questionnaire, and 10 questions assessing the clinician’s impression of the young person after the study.

#### Other parameters

Information regarding demographic characteristics and concomitant treatment is collected. BPD related pathology is measured via the BPD section of the Structured Clinical Interview for DSM-IV Axis II disorders Personality Questionnaire (SCID-II BPD PQ) [51], and the behavior scale of the Borderline Symptom List (BSL-23) [52]. Achievement of developmental milestones is measured via the Developmental Milestones List (DML) [53], which comprises 13 items regarding developmental tasks that young people face when growing up, making up three subscales: personal, social and academic functioning. Items are answered on a 7-point Likert scale. Life experiences are assessed with a shortened version of the Life Experiences Survey (LES) [54]. Psychopathology is measured with a Dutch questionnaire called Screeningsinstrument Psychische Stoornissen (SPSY) [55], which is based on the Strengths and Difficulties Questionnaire (SDQ) [56]. The brief form of the Levels of Personality Functioning Scale 2.0 (LPFS-BF) [57] and Personality Inventory for DSM-5 (PID-5-BF) [58, 59] are included as measures of the Alternative Model of Personality Disorders [60]. Impulsivity is measured with the short version of the UPPS-P impulsive behavior scale [61]. Internalizing problems are measured with the Depression Anxiety Stress Scales (DASS) [62]. Loneliness is assessed with the short version of the Dutch De Jong Gierveld questionnaire [63]. Motivation for change is measured by the Motivation for Youth Treatment Scale (MYTS) [64]. The 11-item version of the Recovering Quality of Life (ReQoL) [65] concerns functioning over the past week, and is answered on a 4-point Likert-scale. During the COVID-19 pandemic (i.e., until March 10, 2023), impact of COVID-19 on daily life was measured via six semi-structured interview questions developed for the study.

### Data management

Participants fill out the questionnaires through an online portal called ‘RoQua’. At GGz Centraal, RoQua is also used for Routine Outcome Monitoring and it is linked to the electronic patient records. Hence, the answers on the questionnaires of this study are available to clinicians through these records. The data is stored in a secure online environment. RoQua sends personalized links via text messages in which the ESM-questionnaires are embedded. RoQua is formally recognized as an instrument to carry out research in the Netherlands and operates in accordance with the European General Data Protection Regulation [66].

Data will be pseudonymized and stored on a secured network, accessible only by members of the research staff. Staff are all subject to medical confidentiality via their employment at the mental healthcare facility. Personally identifiable material, such as study consent forms, will be stored separately from research data files. Data will be stored for 20 years after completion of the trial. Data integrity will be supported by fully computerized procedures, i.e., participants answering all questionnaires via a computer or their smartphone (within the RoQua environment). Hence, the chance of data being recorded incorrectly is improbable.

### Safety considerations

As the PRIMARY study includes young people who self-harm, the participant group can be considered a higher risk population, given the inherent risk of severe bodily damage and the predictive connection of self-harm to suicide attempts [15]. Hence, careful consideration was given to the safety procedures and to imbedding this study within a safe care system. Previous ESM-studies have shown that frequently asking an individual about their symptoms, even suicidal ideation or negative emotions, does not increase the frequency of these symptoms [27, 43, 67, 68]. Furthermore, ESM was considered a promising method in the development of effective interventions for young people who self-harm [38] as it provides young people with the opportunity to monitor and intervene before self-harm occurs [69]. If acute suicidality is identified during a weekly report session or a research assessment, a structured suicide-risk-protocol will be activated. This protocol was developed in line with the Dutch guidelines regarding suicide prevention [70], and involves the RA sharing the information with a clinician at the healthcare institution who will put the necessary interventions in place.

Serious adverse events, such as events that require hospitalization, will be monitored and reported to the Medical Ethical Board Utrecht within 15 days after the sponsor’s first awareness of the event. Serious adverse events that result in death or are life threatening will be reported within seven days of first knowledge. Since serious adverse events are monitored by the Medical Ethical Board, and given the safe care system in which the study is embedded, a Data Monitoring Committee is not required.

### Data analysis plan and power calculations

Analyses that cover effectiveness of PRIMARY will be performed according to a modified version of the *intention-to-treat* principle [71], which entails including all participants with at least one data point post-baseline regardless of their compliance during the study. This way, random selection in the sample will be guaranteed which ensures the most accurate conclusions following the analyses. In the following section, analyses will be outlined for each study objective (i.e., research question) as well as the required sample size to answer the research questions based on relevant power analyses and recent literature^1^. Multiple imputation will be considered if the amount of missing values is non-trivial. The randomization stratification variable of age and gender will be examined as covariates in the analyses described below. Other covariates, such as concomitant treatment, will also be considered.

To examine the effectiveness of PRIMARY, an ANCOVA-test will be used to compare data from Wave 1 and 2 by study group (study objective 1). Specifically, the following variables will be examined: emotion regulation, quality of relationships, and self-harm. To reach a power of 0.80, the power analyses run in G*Power 3.1 [72]^2^ and R [73], with an additional correction for the pre-test as a covariate, indicated a sample size of *n* = 48 per group to be necessary to detect a medium effect size (α = 0.05). Taking into account a drop-out of 10% (based on the feasibility study), a total sample size of *N* = 106 is necessary to answer this research question.

Regarding the effect of PRIMARY on the effectiveness of subsequent specialized treatment (i.e., HYPE), an ANOVA-test repeated measures with a within-between interaction will be applied to Wave 1, 2 and 3 including only those participants receiving HYPE treatment (study objective 2). To reach a power of 0.80, a sample size of *N* = 42-106 is necessary when considering a medium-large effect size (G*Power 3.1) [72]^1^. Taking into account the 10% drop-out from the feasibility study, a sample size of *N* = 46-118 is required (i.e., young people receiving HYPE treatment).

To increase knowledge on underlying processes of self-harming thoughts and behaviors, and adaptive coping strategies on an individual level, a link between these constructs and emotions and social interactions will be examined using ESM data (study objective 3). Given the large number of measurements of the ESM-questionnaire (i.e., five times a day for 28 consecutive days per participant), these data are suitable for a single-case experimental design [74]. This design focusses on the within-component of the data and is useful for investigating individual processes over time. Additionally, these analyses will be repeated on a group-level. Given the novelty of these types of analyses, it is expected that there will be relevant developments regarding statistical models in the upcoming years. Hence, the best applicable analyses will be considered once the data is collected. Currently, Group Iterative Multiple Model Estimation (GIMME) [75] would be an appropriate type of analysis to answer this research question. For GIMME, at least 60 data points are needed from each participant to reach sufficient power [76], i.e., participants with ≥ 43% compliance would be included.

Lastly, to characterize participants’ and clinicians’ evaluation of PRIMARY (study objective 4), descriptive statistics (e.g., means, standard deviations) will be calculated on the relevant questionnaires at Wave 2.

### Ethics and dissemination

Ethical approval for this study was granted by the Medical Ethical Board Utrecht, the Netherlands (NL73936.041.20) on the 15th of July 2020. Important protocol modifications will be amended and submitted to the Medical Ethical Board Utrecht. Careful attention has been devoted to the consideration of participant privacy and confidentiality. Procedures to support privacy and confidentiality were discussed in detail with the legal department of GGz Centraal and are in line with the European and Dutch regulations on privacy [66]. Participants can withdraw their consent and stop their participation in the study at any time without providing a reason. When this is the case, the data that has been collected thus far will be used in the study, unless the participant specifically requests their data to be deleted.

The study’s protocol has been publicly registered at the International Standard Randomized Controlled Trial Number registry (ISRCTN registry; doi: 10.1186/ISRCTN42088538). The final trial dataset belongs to the sponsor (i.e., GGz Centraal). The principal investigator has access to the dataset. The trial protocol, statistical code and other relevant information will be made publicly available on an appropriate archive platform for sharing purposes (e.g., Open Science Framework) alongside the publication of the trials’ results. The dataset will be available to other researchers on request. They can send in a brief research proposal to request access to the relevant data and may report on additional findings from the study.

Major outcomes of the study will be presented in publications in international peer-reviewed journals, at academic congresses, and any other forum in a de-identified form by the principal investigator, members of the steering committee and researchers who have significantly contributed in the execution of the study (e.g., PhD candidates). Authorship eligibility for publications will be based on contribution to the study and specific manuscript, in accordance with the Contributor Roles Taxonomy (CRediT) statement [77]. Dissemination of findings will also commence in plain langue via the sponsors’ website. To promote the dissemination of findings in the clinical field, results will also be published in practice-oriented journals and presented at clinically oriented workshops and symposia.

## Discussion

The aim of the current multicenter randomized controlled trial is to examine the effectiveness of PRIMARY, a smartphone-based intervention for young people who self-harm and are awaiting further care at a mental healthcare institution. Specifically, its effect on emotion regulation and self-harming thoughts and behaviors will be examined. By using the ESM-methodology as both an intervention and measurement tool, the PRIMARY design is new within the field. Specific attention was given to increasing the feasibility of the study by co-designing PRIMARY with young people with lived experience of mental ill-health, including insights from earlier ESM-studies, and by conducting a feasibility study.

This study strives to evaluate an evidence-based, low-cost, structured intervention which can be offered to those seeking help in the future. Furthermore, the ESM-data allows for a detailed examination of the processes underlying self-harm, which will contribute to theoretical knowledge regarding this behavior.

### Trial status

After receiving ethical approval, a feasibility study was conducted between January and September 2021, whereafter adjustments were made to ensure feasibility of the RCT. The first participant was included in the RCT in October 2021. The study is currently ongoing. Data collection is predicted to continue until April 2026.

International trial-registration was uploaded retrospectively on the 26th of October 2022 at the ISRCTN registry (identification code: ISRCTN42088538). This protocol is version 1, dated September 2022.

### Electronic supplementary material

Below is the link to the electronic supplementary material.

Supplementary Material 1

## Data Availability

The datasets generated and/or analyzed during the current study are not publicly available to protect study participant privacy. They are available from the corresponding author on reasonable request. Data are stored at controlled access servers at GGz Centraal.

## Footnotes

1 New developments concerning the outlined statistical analyses and accompanying power analyses led to the adjustment of the necessary sample size in comparison to the ISTRCN registration, where a total of 180 participants was mentioned.

2 G*Power assumes the comparison group to deteriorate over time. However, stability was expected in this group in the current study. Hence, the suitable correction was implemented in G*Power when calculating the sample size needed to answer this research question.

## Declarations

### Protocol version

Issue date: October 2nd, 2023; Protocol version: 01.

### Steering committee

Members: CJH (Principal investigator), MAGvA, OML, AMC, JKB, G. Blom, anonymous young people with lived experience of mental-ill health. The steering committee provided feedback on the design of the study and advice where needed during the implementation. G. Blom supported the young people with lived experience during this process.

## Abbreviations

PRIMARY: PRe-Intervention Monitoring of Affect and Relationships in Youth
ESM: Experience Sampling Method
ISRCTN: International Standard Randomized Controlled Trial Number
DBT: Dialectic Behavioral Therapy
MBT: Mentalization Based Treatment
HYPE: Helping Young People Early
BPD: borderline personality disorder
RCT: randomized controlled trial
SPIRIT: Standard Protocol Items: Recommendations for Interventional Trials
CAT: Cognitive Analytic Therapy
PANAS: Positive And Negative Affect Scale
DERS: Difficulties in Emotion Regulation Scale
NRI-BSV: Network of Relationships Inventory – Behavior Systems Version
UMUX: Usability Metric for User Experience
CSQ-8: Client Satisfaction Questionnaire-8
SCID-II BPD PQ: Structured Clinical Interview for DSM-IV Axis II disorders Personality Questionnaire
BSL-23: Borderline Symptom List
DML: Developmental Milestones List
LES: Life Experiences Survey
SPSY: Screeningsinstrument Psychische Stoornissen
SDQ: Strengths and Difficulties Questionnaire
LPFS-BF: Levels of Personality Functioning Scale 2.0 - Brief Form
PID-5-BF: Personality Inventory for DSM-5 - Brief Form
S-UPPS: short UPPS-P impulsive behavior scale
DASS: Depression Anxiety Stress Scales
MYTS: Motivation for Youth Treatment Scale
ReQoL: Recovering Quality of Life
GDPR: General Data Protection Regulation
ANCOVA: Analysis of Covariance
ANOVA: Analysis of Variance
