# Supplementary material for: A smartphone-based intervention for young people who self-harm (‘PRIMARY’): study protocol for a multicenter randomized controlled trial
Source: BMC Psychiatry. 2023 Nov 14;23:840. doi: 10.1186/s12888-023-05301-x (PMC10647141; doi:10.1186/s12888-023-05301-x)
Supplement: Supplementary file 1 — Supplementary Material 1 [file 12888_2023_5301_MOESM1_ESM.docx]

**Supplemental Material**

**Content Page**

Feasibility study 2

Interview Participants Feasibility Study 5

Model Consent Forms 6

ESM-questionnaire 9

Questions Regarding Self-Harming Thoughts and Behaviors 13

Modified Client Satisfaction Questionnaire (CSQ-8) 14

Additional Questions Regarding Satisfaction PRIMARY 15

Questionnaire for Clinicians 16

Trial Registration Data Set from the World Health Organization 19

SPIRIT Checklist 21

References 27

**Feasibility study**

A feasibility study was conducted between January and September 2021 to optimize the subsequent RCT regarding the PRIMARY intervention, i.e., to inform necessary changes to the intervention and study design. Specifically, attention was given to the feasibility of the procedures in light of recruitment pace and ESM compliance rate. In addition to the measures described in the paper, an interview was conducted with the participants of the feasibility study to gather feedback (also included as Supplemental material). Due to the COVID-19 pandemic restrictions at the time, the assessments were conducted via video call.

**Sample and Design**

Ten young people aged between 12 and 25 years (*M* = 18.20, *SD* = 3.29, 90% female) were recruited from the HYPE program at GGz Centraal between January and September 2021. The participants, and their parents or legal guardians for those aged < 16 years, provided written informed consent. All participants participated in the intervention group and completed the measures as described in the paper.

During the feasibility study, inclusion criteria were: (1) engagement in at least one act of self-harm over the past four weeks, (2) a score of six or higher on the SCID-II, and (3) sufficient understanding and skills in the Dutch language.

On average, participants reported that they had hurt themselves 26 days in the past year (range 1-100). The participants had a mean score of 11.90 (*SD* = 2.13) on the SCID-II BPD PQ.

**Results**

***ESM Compliance***

The participants completed on average 59% (range 27-87%) of the 140 ESM-questionnaires (*M* = 84.40 measurements, *SD* = 26.92). All participants reached an attendance of 100% at the weekly report sessions.

***Participant Feedback***

Participants were moderately positive about the usability of the PRIMARY ESM-element on the UMUX questionnaire (*M,* = 61.11, *SD* = 19.95, scale range 0-100). Forty percent rated five measurements a day as ‘not too much, not too few’. Thirty percent rated the frequency as ‘a bit much’. The majority of the participants rated the duration of four weeks for the intervention as ‘not too long, not too short’ (70%). Furthermore, 70% of the participants would recommend PRIMARY to a friend who needs similar help.

***Recruitment***

Recruitment for the feasibility study was anticipated to be completed within 8 to 12 weeks. However, only two participants were included within this timeframe. Instead, it took 46 weeks to recruit 10 participants.

**Conclusions**

Based on the ESM compliance and feedback, the ESM-questionnaire and schedule (i.e., frequency and duration) were judged as feasible and therefore not changed in the RCT. During the interview, participants reported that it was acceptable, even helpful, that the weekly report sessions were held via video calls, rather than being held in person at the institution. It minimized any interference with their daily life and it saved time. Therefore, it was decided for the RCT to allow participants to choose whether they want to do the assessments at the mental health institution or via video call. Participants reported that the encouraging text messages, sent once a week, did not bother them and sometimes helped them to fill out the ESM-questionnaire more often.

Given the slow inclusion rate during the feasibility study, four changes were made to the design of the RCT. First, the original inclusion criterion regarding the timeframe for self-harm was increased from the past four weeks to the past year. This change was made based after reconsidering the literature, in which one act of self-harm in the past year emerged as a signal for a heightened risk for the recurrence of the behavior (Fitzpatrick & Kuo, 2021). Given the aim of PRIMARY is to intervene as early as possible, widening the inclusion to the past year was in line with this aim. Second, the original inclusion criterion of a score of six or higher on the SCID-II BPD PQ was discontinued. This criterion was originally included to select young people at risk for developing BPD. However, new insights revealed self-harm already to be important precursor of BPD (Kaess et al., 2014). The SCID-II BPD PQ is still included in the RCT in order to characterize the sample. Third, the recruitment procedure was modified to enhance participant engagement with the researchers (e.g., contact with a research assistant in addition to receiving the information letter). Fourth, additional recruitment and intervention sites (i.e., other divisions of GGz Centraal and Mondriaan) were employed, making the RCT multicenter.

**Interview Participants Feasibility Study**

1. What did you like about the daily questionnaires?
2. What did you dislike about the daily questionnaires?
3. Did you manage to fill out the questionnaires several times a day? Why/why not? What could have helped you to fill out more questionnaires?
4. Did you experience any technical issues? If yes, what kind of issues?
5. What did you like about the weekly report sessions?
6. What did you dislike about the weekly report sessions?
7. What did you think about the fact that the weekly report sessions were online? Did you experience any technical issues?
8. Did you miss any of the weekly report sessions? If yes, what was the cause of this? What could have helped you to attend more weekly report sessions?
9. What did you like about the encouraging text messages?
10. What did you dislike about the encouraging text messages?
11. Do you have any other remarks?

**Model Consent Forms**

**Consent form participant**

- I have understood the information. I was able to ask questions. My questions were answered.
- I have had enough time to decide whether I want to participate.
- I know I am not obliged to participate.
- I understand that I can always quit when I do not want to participate anymore.
- I give consent to inform my clinician in charge that I participate in this study and in case of a crisis situation.
- I give consent to my clinicians having access to my research data.
- In case I revoke my consent, I understand that the research data that has already been collected will still be processed for scientific research.
- I give consent for the researchers to have access to my details in the electronic patient record. The researchers will only use the electronic patient record in the context of the current study and do not access other information in the electronic patient record.

**I want to participate in this study.**

Name participant:

Signature: Date : __ / __ / __

**If you are aged < 16 years, your parent(s) or legal guardian also have to give consent your participation in this study. If you are you older than 16 years, you can decide on your own if you want to participate.**

**At GGz Centraal, young people also fill out other questionnaires to track the progress of their treatment. Can we ask for your consent to use this data for scientific research in the future?**

- **Yes**
- **No**

**Consent form parent(s)/legal guardian**

I am asked to give consent for my child to participate in this study:

- I have read the information letter. I understand this information and I was able to ask questions. My questions were sufficiently answered. I have had enough time to decide whether I want my child to participate.
- I know that my child’s participation is completely voluntarily. I know that I can revoke my consent any time without providing a reason.
- I give consent for informing the clinician in charge that my child participates in this study and in case of a crisis situation.
- I give consent that clinicians have access to the research data of my child.
- When I revoke my consent, I understand that the research data that has already been collected will still be processed for scientific research.
- I give consent for the researchers to gain access to my child’s details in the electronic patient record. The researchers will only use the electronic patient record in the context of the study and do not access other information in the electronic patient record.

**I give consent for my child’s participation in this study.**

Name parent 1/legal guardian:

Signature: Date : __ / __ / __

Name parent 2/legal guardian:

Signature: Date : __ / __ / __

**ESM-questionnaire**

Questionnaires are sent 5 times a day, for 28 consecutive days.

- = 1 answer possible
- = more answers possible

**General**

1. What are you doing right now?

- Nothing
- Working
- Studying/at school
- Working out/walking/cycling
- Hobby (e.g., making music)
- Household chores
- Self-care (e.g., showering, make-up)
- Relaxing
- Television/YouTube/Internet
- Social media
- Gaming
- Talking to someone
- Getaway/outing (e.g., to the city centre, concert)
- Eating or drinking
- Traveling
- Other

1. Who are you with, right now?

- Nobody
- My partner
- Parent(s)
- Brother/sister
- Other family member
- Best friend
- Friends
- Acquaintance(s)
- Stranger(s)
- Professional (e.g., teacher, caregiver)
- Other

1. Who are you with on social media, right now?

- Nobody
- My partner
- Parent(s)
- Brother/sister
- Other family member
- Best friend
- Friends
- Acquaintance(s)
- Stranger(s)
- Professional (e.g., teacher, caregiver)
- Other

**Social Interactions**

1. To what extent do you feel supported right now?


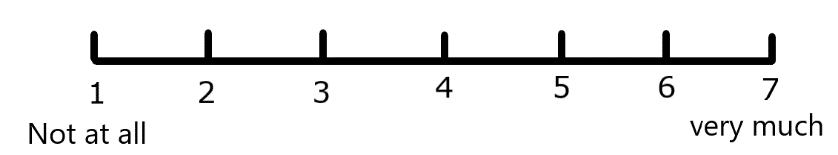


1. To what extent do you experience conflict right now?


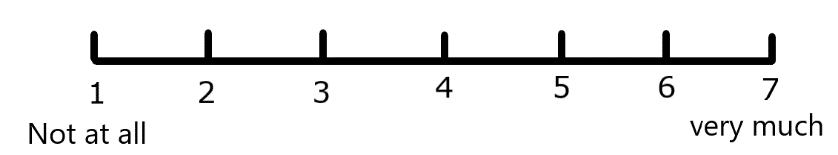


**Emotion(regulation)**

*Questions 6 - 20 are answered using the following scale:*


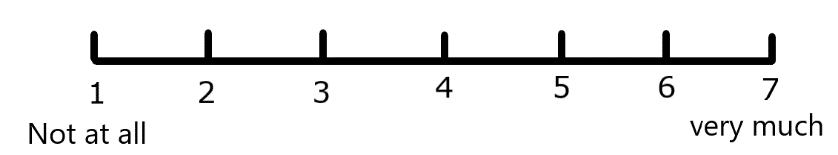


At this moment, to what extent do you feel:

1. Relaxed
2. Unhappy
3. Happy
4. Lonely
5. Annoyed
6. Confident
7. Disappointed
8. Energetic
9. Angry
10. Sad
11. Enthousiastic
12. Nervous
13. Tired
14. Empty
15. Satisfied
16. Since the last beep, did you do something to improve your mood?

- Not applicable, it was okay for me
- I tried to think of something else
- I looked for a distraction (e.g., working out, hobby, listening to music)
- I looked for support (e.g., from a friend, family member or a health care professional)
- I expressed my emotions (e.g., crying or screaming)
- I tried to solve a problem
- I didn’t do anything
- I thought about hurting myself
- I did something else: […]

*Only when ‘I thought about hurting myself’ is checked, question 22 appears.*

1. Since the last beep, did you intentionally hurt yourself?

- No
- Yes

**Substance Use**

1. What did you use since the last beep?
   - Coffee
   - Energy drink (red bull, bullit, etc.)
   - Alcohol
   - Cigarettes
   - Cannabis, nitrous oxide or other drugs
   - Medication
   - None of the above

**Functioning**

*The last ESM-questionnaire of every day contains two additional questions:*

1. Did you break any rules today? (e.g., stealing, breaking something on purpose)

- No
- Yes

1. To what extent did you manage to do things the way you planned?


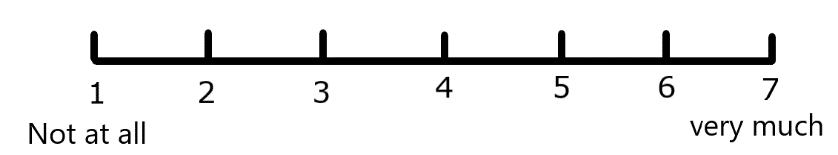


**Questions Regarding Self-Harming Thoughts and Behaviors**

1. Have you ever intentionally hurt yourself (e.g., cutting, scratching, burning, or hitting)?

- Yes
- No

2. How many times in the past four weeks have you intentionally hurt yourself (e.g., cutting, scratching, burning, or hitting)?

3. How many days in the past year have you intentionally hurt yourself?

4. How many times in the past year did you need medical care after you intentionally hurt yourself (e.g., a visit to a doctor or emergency room)?

5. How old were you when you intentionally hurt yourself for the first time?

**Modified Client Satisfaction Questionnaire (CSQ-8)**

1. To what extent has our program met your needs?

- Almost all of my needs have been met
- Most of my needs have been met
- Only a few of my needs have been met
- None of my needs have been met

1. In an overall, general sense, how satisfied are you with the service you received?

- Very satisfied
- Mostly satisfied
- Indifferent or mildly dissatisfied
- Quite dissatisfied

1. Have the services you received helped you to deal more effectively with your problems?

- Yes, they helped a great deal
- Yes, they helped somewhat
- No, they really didn’t help
- No, they seemed to make things worse

1. If a friend were in need of similar help, would you recommend our program to him/her?

- No, definitely not
- No, I don’t think so
- Yes, I think so
- Yes, definitely

**Additional Questions Regarding Satisfaction PRIMARY**

1. I found the number of assessments (5) a day:

- Very low
- Low
- Good
- High
- Very high

1. I found using PRIMARY for four weeks:

- Very short
- Short
- Good
- Long
- Very long

**Questionnaire for Clinicians**

1. This young person can recognize several of their emotions

- Strongly agree
- Agree
- Agree nor disagree
- Disagree
- Strongly disagree

1. This young person has insight in the connection between emotions and situations
   - Strongly agree
   - Agree
   - Agree nor disagree
   - Disagree
   - Strongly disagree
2. This young person has several adaptive ways to cope with his/her emotions
   - Strongly agree
   - Agree
   - Agree nor disagree
   - Disagree
   - Strongly disagree
3. This young person seeks support in his/her social network
   - Strongly agree
   - Agree
   - Agree nor disagree
   - Disagree
   - Strongly disagree
4. This young person is motivated for treatment
   - Strongly agree
   - Agree
   - Agree nor disagree
   - Disagree
   - Strongly disagree
5. I examined the answers on the PRIMARY-questionnaire of this young person before the appointment.
   - Yes
   - No
6. The answers on the PRIMARY-questionnaires gave me useful information about this young person.
   - Strongly agree
   - Agree
   - Agree nor disagree
   - Disagree
   - Strongly disagree

*Regarding participants in the intervention group:*

1. I examined the graphical reports of this young person before I the appointment.
   - Yes
   - No
2. The graphs gave me useful information about this young person.
   - Strongly agree
   - Agree
   - Agree nor disagree
   - Disagree
   - Strongly disagree

**Trial Registration Data Set from the World Health Organization**

| Data category | Information |
| --- | --- |
| Primary registry and trial identification number | ISTRCTN; ISRCTN42088538 |
| Date of registration in primary registry | October 26, 2022 |
| Secondary identifying numbers | Protocol/serial number 636310013 |
| Source(s) of monetary or material support | ZonMw |
| Sponsor | GGz Centraal |
| Contact for public queries | Dr. Christel Hessels; c.hessels@ggzcentraal.nl |
| Contact for scientific queries | Dr. Christel Hessels; c.hessels@ggzcentraal.nl |
| Public title | The PRIMARY study: a study into the effectiveness of daily registration of emotions, social context, activities and weekly feedback for young people who self-harm |
| Scientific title | Pre-Intervention Monitoring of Affect and Relationships in Youth: a Randomized Controlled Trial (RCT) into the effectiveness of an Ecological Sampling Method (ESM) intervention in combination with weekly feedback for young people who self-harm |
| Countries of recruitment | The Netherlands |
| Health condition(s) or problem(s) studied | Self-harm; borderline personality pathology |
| Intervention(s) | PRIMARY |
| Key inclusion and exclusion criteria | Inclusion criteria: ages 12-25; self-harm in past year; sufficient skills in Dutch language.  Exclusion criteria: NA |
| Study type | Interventional; randomized controlled trial; parallel assignment |
| Date of first enrolment | October 07, 2021 |
| Target sample size | 180 |
| Recruitment status | Recruiting |
| Primary outcome(s) | Self-harm, emotion regulation, quality of relationships |
| Key secondary outcomes | Usability of PRIMARY, satisfaction with PRIMARY |


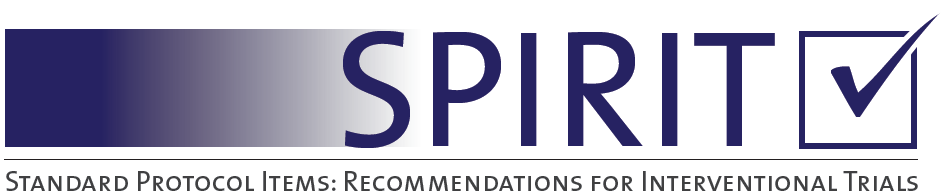


SPIRIT 2013 Checklist: Recommended items to address in a clinical trial protocol and related documents*

| Section/item | Item No | Description | Addressed on page number |
| --- | --- | --- | --- |
| **Administrative information** | | |  |
| Title | 1 | Descriptive title identifying the study design, population, interventions, and, if applicable, trial acronym | ______1______ |
| Trial registration | 2a | Trial identifier and registry name. If not yet registered, name of intended registry | ______2______ |
|  | 2b | All items from the World Health Organization Trial Registration Data Set | Supplemental material (p. 19-20) |
| Protocol version | 3 | Date and version identifier | _____10_____ |
| Funding | 4 | Sources and types of financial, material, and other support | _____10_____ |
| Roles and responsibilities | 5a | Names, affiliations, and roles of protocol contributors | ___1; 10_____ |
|  | 5b | Name and contact information for the trial sponsor | _____10_____ |
|  | 5c | Role of study sponsor and funders, if any, in study design; collection, management, analysis, and interpretation of data; writing of the report; and the decision to submit the report for publication, including whether they will have ultimate authority over any of these activities | _____10_____ |
|  | 5d | Composition, roles, and responsibilities of the coordinating centre, steering committee, endpoint adjudication committee, data management team, and other individuals or groups overseeing the trial, if applicable (see Item 21a for data monitoring committee) | _____10_____ |
| Introduction |  |  |  |
| Background and rationale | 6a | Description of research question and justification for undertaking the trial, including summary of relevant studies (published and unpublished) examining benefits and harms for each intervention | _____2-3_____ |
|  | 6b | Explanation for choice of comparators | ______3_____ |
| Objectives | 7 | Specific objectives or hypotheses | ______3_____ |
| Trial design | 8 | Description of trial design including type of trial (eg, parallel group, crossover, factorial, single group), allocation ratio, and framework (eg, superiority, equivalence, noninferiority, exploratory) | ______3______ |
| Methods: Participants, interventions, and outcomes | | |  |
| Study setting | 9 | Description of study settings (eg, community clinic, academic hospital) and list of countries where data will be collected. Reference to where list of study sites can be obtained | ______3-4_____ |
| Eligibility criteria | 10 | Inclusion and exclusion criteria for participants. If applicable, eligibility criteria for study centres and individuals who will perform the interventions (eg, surgeons, psychotherapists) | _____3-4_____ |
| Interventions | 11a | Interventions for each group with sufficient detail to allow replication, including how and when they will be administered | ______5_____ |
|  | 11b | Criteria for discontinuing or modifying allocated interventions for a given trial participant (eg, drug dose change in response to harms, participant request, or improving/worsening disease) | _____7-8_____ |
|  | 11c | Strategies to improve adherence to intervention protocols, and any procedures for monitoring adherence (eg, drug tablet return, laboratory tests) | _____3-4_____ |
|  | 11d | Relevant concomitant care and interventions that are permitted or prohibited during the trial | ______4______ |
| Outcomes | 12 | Primary, secondary, and other outcomes, including the specific measurement variable (eg, systolic blood pressure), analysis metric (eg, change from baseline, final value, time to event), method of aggregation (eg, median, proportion), and time point for each outcome. Explanation of the clinical relevance of chosen efficacy and harm outcomes is strongly recommended | _____6-7_____ |
| Participant timeline | 13 | Time schedule of enrolment, interventions (including any run-ins and washouts), assessments, and visits for participants. A schematic diagram is highly recommended (see Figure) | ___Figure 1___ |
| Sample size | 14 | Estimated number of participants needed to achieve study objectives and how it was determined, including clinical and statistical assumptions supporting any sample size calculations | ______8_____ |
| Recruitment | 15 | Strategies for achieving adequate participant enrolment to reach target sample size | 4; Supplemental material (p. 2-4) |
| **Methods: Assignment of interventions (for controlled trials)** | | |  |
| Allocation: |  |  |  |
| Sequence generation | 16a | Method of generating the allocation sequence (eg, computer-generated random numbers), and list of any factors for stratification. To reduce predictability of a random sequence, details of any planned restriction (eg, blocking) should be provided in a separate document that is unavailable to those who enrol participants or assign interventions | ______4______ |
| Allocation concealment mechanism | 16b | Mechanism of implementing the allocation sequence (eg, central telephone; sequentially numbered, opaque, sealed envelopes), describing any steps to conceal the sequence until interventions are assigned | ______4______ |
| Implementation | 16c | Who will generate the allocation sequence, who will enrol participants, and who will assign participants to interventions | ______4______ |
| Blinding (masking) | 17a | Who will be blinded after assignment to interventions (eg, trial participants, care providers, outcome assessors, data analysts), and how | ______4______ |
|  | 17b | If blinded, circumstances under which unblinding is permissible, and procedure for revealing a participant’s allocated intervention during the trial | ______NA_____ |
| **Methods: Data collection, management, and analysis** | | |  |
| Data collection methods | 18a | Plans for assessment and collection of outcome, baseline, and other trial data, including any related processes to promote data quality (eg, duplicate measurements, training of assessors) and a description of study instruments (eg, questionnaires, laboratory tests) along with their reliability and validity, if known. Reference to where data collection forms can be found, if not in the protocol | 6-7; Table 1; Supplemental material (p. 9-18) |
|  | 18b | Plans to promote participant retention and complete follow-up, including list of any outcome data to be collected for participants who discontinue or deviate from intervention protocols | _____4,9____ |
| Data management | 19 | Plans for data entry, coding, security, and storage, including any related processes to promote data quality (eg, double data entry; range checks for data values). Reference to where details of data management procedures can be found, if not in the protocol | ______7_____ |
| Statistical methods | 20a | Statistical methods for analysing primary and secondary outcomes. Reference to where other details of the statistical analysis plan can be found, if not in the protocol | ______8_____ |
|  | 20b | Methods for any additional analyses (eg, subgroup and adjusted analyses) | ______8_____ |
|  | 20c | Definition of analysis population relating to protocol non-adherence (eg, as randomised analysis), and any statistical methods to handle missing data (eg, multiple imputation) | ______8_____ |
| **Methods: Monitoring** | | |  |
| Data monitoring | 21a | Composition of data monitoring committee (DMC); summary of its role and reporting structure; statement of whether it is independent from the sponsor and competing interests; and reference to where further details about its charter can be found, if not in the protocol. Alternatively, an explanation of why a DMC is not needed | ______8_____ |
|  | 21b | Description of any interim analyses and stopping guidelines, including who will have access to these interim results and make the final decision to terminate the trial | ______NA_____ |
| Harms | 22 | Plans for collecting, assessing, reporting, and managing solicited and spontaneously reported adverse events and other unintended effects of trial interventions or trial conduct | ______8______ |
| Auditing | 23 | Frequency and procedures for auditing trial conduct, if any, and whether the process will be independent from investigators and the sponsor | ______NA_____ |
| Ethics and dissemination | | |  |
| Research ethics approval | 24 | Plans for seeking research ethics committee/institutional review board (REC/IRB) approval | ______9______ |
| Protocol amendments | 25 | Plans for communicating important protocol modifications (eg, changes to eligibility criteria, outcomes, analyses) to relevant parties (eg, investigators, REC/IRBs, trial participants, trial registries, journals, regulators) | ______9______ |
| Consent or assent | 26a | Who will obtain informed consent or assent from potential trial participants or authorised surrogates, and how (see Item 32) | ______5______ |
|  | 26b | Additional consent provisions for collection and use of participant data and biological specimens in ancillary studies, if applicable | _____NA______ |
| Confidentiality | 27 | How personal information about potential and enrolled participants will be collected, shared, and maintained in order to protect confidentiality before, during, and after the trial | ______7______ |
| Declaration of interests | 28 | Financial and other competing interests for principal investigators for the overall trial and each study site | _____10______ |
| Access to data | 29 | Statement of who will have access to the final trial dataset, and disclosure of contractual agreements that limit such access for investigators | _____9-10____ |
| Ancillary and post-trial care | 30 | Provisions, if any, for ancillary and post-trial care, and for compensation to those who suffer harm from trial participation | _____7-8____ |
| Dissemination policy | 31a | Plans for investigators and sponsor to communicate trial results to participants, healthcare professionals, the public, and other relevant groups (eg, via publication, reporting in results databases, or other data sharing arrangements), including any publication restrictions | ______9_____ |
|  | 31b | Authorship eligibility guidelines and any intended use of professional writers | ______9_____ |
|  | 31c | Plans, if any, for granting public access to the full protocol, participant-level dataset, and statistical code | _____9-10____ |
| Appendices |  |  |  |
| Informed consent materials | 32 | Model consent form and other related documentation given to participants and authorised surrogates | Supplemental material (p. 6-8) |
| Biological specimens | 33 | Plans for collection, laboratory evaluation, and storage of biological specimens for genetic or molecular analysis in the current trial and for future use in ancillary studies, if applicable | _____NA______ |

*It is strongly recommended that this checklist be read in conjunction with the SPIRIT 2013 Explanation & Elaboration for important clarification on the items. Amendments to the protocol should be tracked and dated. The SPIRIT checklist is copyrighted by the SPIRIT Group under the Creative Commons “[Attribution-NonCommercial-NoDerivs 3.0 Unported](http://www.creativecommons.org/licenses/by-nc-nd/3.0/)” license.

**References**

Fitzpatrick, S., & Kuo, J. R. (2021). Predicting the effectiveness of engagement and disengagement emotion regulation based on emotional reactivity in borderline personality disorder. *Cognition & Emotion*, 1–19. https://doi.org/10.1080/02699931.2021.2018291

Kaess, M., Brunner, R., & Chanen, A. (2014). Borderline personality disorder in adolescence. *Pediatrics*, *134*(4), 782–793. https://doi.org/10.1542/peds.2013-3677
